# Intra-operative intravenous fluid restriction reduces perioperative red blood cell transfusion in elective cardiac surgery, especially in transfusion-prone patients: a prospective, randomized controlled trial

**DOI:** 10.1186/1749-8090-5-7

**Published:** 2010-02-24

**Authors:** George Vretzakis, Athina Kleitsaki, Konstantinos Stamoulis, Metaxia Bareka, Stavroula Georgopoulou, Menelaos Karanikolas, Athanasios Giannoukas

**Affiliations:** 1 Cardiac Anesthesia Unit, Department of Anesthesiology, University Hospital of Larissa, Greece; 2 Department of Anaesthesiology and Critical Care, University of Patras School of Medicine, Greece; 3 Department of Vascular Surgery, University Hospital of Larissa, Greece

## Abstract

**Background:**

Cardiac surgery is a major consumer of blood products, and hemodilution increases transfusion requirements during cardiac surgery under CPB. As intraoperative parenteral fluids contribute to hemodilution, we evaluated the hypothesis that intraoperative fluid restriction reduces packed red-cell (PRC) use, especially in transfusion-prone adults undergoing elective cardiac surgery.

**Methods:**

192 patients were randomly assigned to restrictive (group A, 100 pts), or liberal (group B, 92 pts) intraoperative intravenous fluid administration. All operations were conducted by the same team (same surgeon and perfusionist). After anesthesia induction, intravenous fluids were turned off in Group A (fluid restriction) patients, who only received fluids if directed by protocol. In contrast, intravenous fluid administration was unrestricted in group B. Transfusion decisions were made by the attending anesthesiologist, based on identical transfusion guidelines for both groups.

**Results:**

137 of 192 patients received 289 PRC units in total. Age, sex, weight, height, BMI, BSA, LVEF, CPB duration and surgery duration did not differ between groups. Fluid balance was less positive in Group A. Fewer group A patients (62/100) required transfusion compared to group B (75/92, p < 0.04). Group A patients received fewer PRC units (113) compared to group B (176; p < 0.0001). Intraoperatively, the number of transfused units and transfused patients was lower in group A (31 u in 19 pts vs. 111 u in 62 pts; p < 0.001). Transfusions in ICU did not differ significantly between groups. Transfused patients had higher age, lower weight, height, BSA and preoperative hematocrit, but no difference in BMI or discharge hematocrit. Group B (p < 0.005) and female gender (p < 0.001) were associated with higher transfusion probability. Logistic regression identified group and preoperative hematocrit as significant predictors of transfusion.

**Conclusions:**

Our data suggest that fluid restriction reduces intraoperative PRC transfusions without significantly increasing postoperative transfusions in cardiac surgery; this effect is more pronounced in transfusion-prone patients.

**Trial registration:**

NCT00600704, at the United States National Institutes of Health.

## Background

Cardiac surgery is a major blood product consumer. Data from many studies suggest that blood transfusions are associated with increased morbidity and mortality in cardiac surgery [1,2]. However, a recent large observational study did not show an association between moderate (≤6 units) blood product exposure and reduced long-term survival [3]. As the risk of transfusion-associated adverse outcomes may depend on the amount of transfusion [4], reduction of blood transfusions is considered a relevant, important goal in cardiac surgery.

During cardiac operations under CPB, two concurrent events, namely blood loss and red blood cell dilution due to positive fluid balance result in precipitous hematocrit drop and need for allogeneic blood. Hemodilution has been identified as a major factor influencing the decision to transfuse. Likewise, several variables associated with total red cell mass, such as preoperative anemia, female gender and small body size, are independent predictors of transfusion in cardiac surgery [5-8]. Existing guidelines underline the importance of limiting hemodilution, applying blood salvage techniques and using alternative therapies for transfusion and blood conservation [7].

Surprisingly, data on the impact of intraoperative parenteral fluid restriction on transfusion needs are very limited. Recently, we published a RCT involving 130 pts operated for CABG under CPB supported by reinfusion of washed shed blood from thoracic cavities, and reported significant reduction of intraoperative PRC transfusions with a restrictive parenteral fluid protocol [9]. However, as only a small proportion of cardiac surgery patients are "transfusion-prone" (as defined by low preoperative hematocrit, female sex, or small BSA) our earlier study did not have adequate power to evaluate the role of fluid restriction on patients prone to transfusion. In contrast, the present study included a higher number of patients, and had adequate power for investigating the impact of perioperative intravenous fluid restriction on red blood cell transfusions not only in cardiac surgery patients in general, but also in the subset of patients who are considered transfusion-prone.

## Methods

### Patient selection and anesthesia

This prospective study was conducted in our University Hospital over a 20-month period, after approval from the Institution Ethics committee, and written informed consent was obtained from all patients before entering the study.

Inclusion criteria were elective cardiac surgery under CPB and ages 18 - 85. Exclusion criteria were emergency or re-do operations, operations starting after 18.00, recent administration of TPA or other thrombolytic medications, pre-existing hematologic disease or coagulation abnormality, advanced cirrhosis, renal failure, preoperative blood product transfusion, combined cardiac and carotid surgery and operations with minimal extracorporeal flow (surgery of ascending aorta) or circulatory arrest.

All patients received standardized anesthesia and intraoperative care, and were operated by the same team (same surgeon, assistant and perfusionist) under standardized conditions (same operating room and setting) with CPB and intra-operative cell salvage. Acute normovolemic hemodilution and retrograde autologous priming of the CPB circuit were not used in any patient. Antiplatelet medications (except aspirin) were discontinued at least 72 hours before surgery. Pharmacologic agents used to decrease blood loss in cardiac surgery (such as aprotinin, aminocaproic acid or tranexamic acid) were not used in any patient.

Monitoring included 5-lead ECG, ST-segment analysis, mixed venous oximetry plus continuous cardiac output recording (*Oximetry TD catheter, Edwards Lifesciences, Germany*), bispectral index (*BIS/XP, Aspect Medical Systems, USA*) and near-infrared spectroscopy to asses cerebrovascular hemoglobin oxygen saturation (*INVOS 5100, Somanetics, USA*).

All patients received total intravenous anesthesia with propofol and remifentanil. Neuromuscular blockade was maintained with cis-atracurium. The CPB pump and tubing (*Stockert SIII, Germany; circuit: Custom Pack, Dideco, Italy*) were primed with 1400 - 2000 mls of crystalloid, based on patient somatometric characteristics. Anticoagulation was achieved with heparin 300 IU/kg of body weight and ACT > 400 s was required before initiating CPB. Pump flow was 2.3-2.5 liter/min/m^2^. All patients received antegrade cardioplegia. Isolated CABG patients were operated under mild passive hypothermia down to 33.5-34.0°C, while systemic drift to 32.0°C was applied on all other patients. The lowest bladder temperatures recorded during CPB were not different between groups (34.58 ± 0.66°C in group A vs. 34.55 ± 0.57°C in group B). Most CABG patients received one internal mammary artery graft plus saphenous veins grafts. Active rewarming to 37.5°C bladder temperature and proper cardiac reperfusion were applied on all patients. After weaning from CPB, protamine 3 mg/kg was given to neutralize heparin. Remaining CPB circuit blood together with blood saved from the operation field was washed, centrifuged (*Electa, Dideco, Italy*) and re-transfused. Red cell salvage continued until the operation finished. Postoperatively all patients were admitted to the ICU, and the same hypnotic-analgesic regimen continued. Criteria for weaning from mechanical ventilation included hemodynamic stability with minimal or no cathecholamine support, absence of significant dysrhythmias, absence of major bleeding, core body temperature > 36°C, proper level of consciousness and acceptable blood gases with good respiratory mechanics. Postoperative pain was controlled with intravenous morphine infusion. Patients transferred to the ward when their clinical condition and laboratory findings were acceptable.

### Study protocol

Surgeon, assistants, perfusionist and ICU personnel were not informed about the study. Anesthesiologists knew there was an ongoing study, but were not informed about the scope and aims of the study. Perfusionists followed common guidelines for cell saver use. Patients meeting inclusion criteria were randomly (using computer-generated numbers) allocated to either group A (restrictive protocol) or group B (control, IV fluid administration "as usual", based on all available hemodynamic data).

The following protocol was applied in group A: Intravenous (IV) fluids before CPB were limited to 500 ml. Peripheral IV lines were connected to hydroxyethylstarch (*Voluven, 6% HES 130/0.4, Fresenius Kabi, France*) and were turned off after central line placement. However, IV fluids were given quickly (within 3-5 minutes) in 50 ml increments when necessary. Anesthetic and inotropic or vasoactive solutions were double-concentrated and administered proximally through the central venous line without a "carrier" fluid infusion. Blood aspirated for sampling was re-infused and excessive line flashing was avoided. Before CPB, hemodynamic instability was managed according to the following algorithm:

A) for MAP < 55 mmHg with SvO_2 _> 75%, INVOS > 60% and BIS < 35 ⇒ titration of anesthetic drugs [*]

B) for MAP <55 mmHg with SvO_2 _> 75%, INVOS > 60% and BIS > 35 ⇒ vasoconstrictor [*]

C) for SvO_2 _< 75%, PCWP ≥ 16 mmHg and heart rate < 90 b/min ⇒ dobutamine

D) for SvO_2 _< 75% and heart rate < 40 b/min ⇒ pacing via epicardial electrode

[*] regardless of filling pressures

After applying the above corrective measures, each anesthesiologist was free to re-evaluate the patient and act according to his/her judgment for any other scenario.

Patients allocated in group B, received Ringer's Lactate solution through their peripheral IV line; drugs were diluted as usual and administered together with a "carrier" infusion at 40 ml/h. Anesthesiologists did not have to follow any specific fluid administration protocol, except for intraoperative PRC transfusion. Access to BIS and INVOS data was unrestricted, and anesthesiologists were free to manage the patient based on their judgment. In both groups, peripheral tissue perfusion/oxygenation was evaluated throughout the procedure, using all available hemodynamic data, including mixed venous oxygen saturation.

### Indications for perioperative PRC transfusion

Perioperative transfusion decisions were made by the attending anesthesiologist, based on the following hematocrit-based rules: During AOX, allogeneic blood was not given if hematocrit was >21%. For values less than 17%, one unit of PRC was transfused. When hematocrit was between 17-21%, anesthesiologists were free to act based on their judgment when treating group B patients. In contrast, when treating fluid-restricted (group A) patients, anesthesiologists were expected to take INVOS values into consideration when deciding about transfusions, as follows: If mean INVOS value from both hemispheres was less than 60 or had decreased by 20% or more, compared to mean value during pulmonary artery catheter insertion, the patient was transfused.

In both groups, after AOX removal and before weaning from CPB (usually near completion of the last proximal anastomosis or during cardiac reperfusion), PRCs were transfused for hematocrit less than 21%. After weaning from CPB and re-transfusion of salvaged blood, patients were transfused for hematocrit ≤24%. In the ICU, patients were transfused for hematocrit ≤24%, while transfusion decisions for hematocrit values between 24-30% were evaluated in a multimodal manner.

### Data collection and statistical analysis

Power analysis for sample size estimation was based on the following assumptions: The total number of PRC units transfused during hospital stay is the main outcome. Mean value of PRC transfusions during hospital stay is 3 units, Standard Deviation is 2 units, and reducing transfusions by one PRC unit is a clinically meaningful improvement compared to standard practice. These assumptions are consistent with data from our institution and also with published data [10]. Based on these assumptions, the study requires 60 patients per group, when α is set at 0.05 and power (1-β) is set at 0.8. However, we decided to enroll up to 100 patients per group, to allow for patient attrition or missing data, and also in order to look for differences with regards to transfusion between patient subgroups.

Total IV fluid volume administered and urine produced before CPB, during CPB and from CPB termination to the end of surgery were recorded for each patient. Priming and cardioplegic solution volumes, additional fluid given during CPB, hemofiltration volumes and pump residual volumes were also recorded. Hematocrit values were recorded preoperatively, after arterial line placement, after anesthesia induction, 10 minutes after CPB started, before CPB termination, at the end of surgery, 6 and 12 hours after ICU admission and before discharge from the hospital. BMI and BSA were calculated with standard formulas. Based on body weight and gender, net erythrocyte volume loss from the day before surgery until hospital discharge, and erythrocyte volume of transfused PRC units were calculated for each patient for the entire hospitalization. Data were stored electronically in Excel and were analyzed with SPSS 15.0 for Windows (SPSS Inc, Chicago, IL).

Continuous data normality was tested with the Kolmogorov-Smirnov test (Lilliefors significant correction) and Shapiro-Wilk test. Demographic and clinical patient characteristics were compared between groups using chi-square test for categorical data and Student's two-tailed t-test for continuous data. "Transfusion" was treated as a dichotomous variable, dividing patients in two subgroups: those who did and those who did not receive PRC transfusions. The association between group assignment (fluid restriction vs. liberal fluids) and gender with transfusion was evaluated with Pearson chi-square and Fisher's exact tests. The association of age, weight, height, BMI, BSA, preoperative Hct and discharge Hct with transfusion were tested with parametric (independent samples T-test) and non parametric (Mann-Whitney U) analyses. P-values < 0.05 were considered significant for all tests. Finally, a logistic regression model was constructed, to evaluate the association of all the above variables with probability of PRC transfusion using the Nagelkerke R^2 ^and Cox & Snell R^2 ^tests.

## Results

Prospectively 192 cardiac surgery patients were randomly assigned to group A (100 patients, restrictive IV fluid administration protocol) or group B (92 patients, liberal IV fluid administration). Baseline demographic and clinical characteristics did not differ significantly between groups (Table 1).

**Table 1:** Demographic, clinical and transfusion data by patient group

Variable	Group A (fluid restriction)	Group B (liberal fluid administration)
Number of pts, n	100	92
Age (years)	66.0 ± 7.9	65.5 ± 8.3
Female gender, n (%)	17 (17.0%)	16 (17.4%)
Weight (kg)	77.2 ± 11.5	75.5 ± 10.6
Height (cm)	167.0 ± 7.8	168.0 ± 7.7
BMI	27.6 ± 3.5	26.7 ± 3.1
BSA (m^2^)	1.84 ± 0.17	1.84 ± 0.16
NYHA I-II, n (%)	57 (57.0%)	55 (59.8%)
NYHA III-IV, n (%)	43 (43.0%)	37 (40.2%)
LVEF (%)	50.2 ± 10.2	48.6 ± 12.1
Diabetes , n (%)	21 (21.0%)	20 (21.7%)
COPD, n (%)	14 (14.0%)	12 (13.0%)
Preop. Hct (%)	40.2 ± 4.42	40.6 ± 3.87
CABG, n (%)	88 (88.0%)	79 (85.9%)
Number of grafts	2.8 ± 0.6	2.7 ± 0.6
AVR + CABG	4 (4%)	4 (4.3%)
AVR	5 (5%)	6 (6.5%)
MVR	3 (3%)	2 (2.1%)
ASD Repair	0 (0%)	1 (1.1%)
CPB time (min)	96.9 ± 22.6	93.1 ± 20.0
AOX (min)	69.2 ± 20.0	67.9 ± 19.2
Operation time (min)	243 ± 49.4	236 ± 47.1

PRC transfused (total)	113 (1.13 ± 1.15*)	176 (1.91 ± 1.35) ◇◇
PRC transfused in OR, n (mean ± SD)	31 (0.31 ± 0.71*)	111 (1.21 ± 3.15) ◇◇
PRC transfused in ICU, n (mean ± SD)	82 (0.82 ± 0.98*)	65 (0.71 ± 0.88)
Transfused pts, n (%)	62 (62.0%)	75 (81.5%) ◇◇
Transfused pts in OR, n (%)	19 (19.0%)	62 (67.4%) ◇◇
Transfused pts in ICU, n (%)	51 (51.0%)	42 (45.7%)
PRC/pt transfused in OR (mean ± SD)	1.63 ± 0.68 **	1.79 ± 0.70
PRC/pt transfused in ICU (mean ± SD)	1.61 ± 0.78 **	1.55 ± 0.63
Females transfused, n (%)	16 (94.1%)	16 (100.0%)
Pts receiving ≥ 4 PRC u (OR + ICU)	2 (2.0%)	13 (14.1%) ◇

* denotes mean ± SD for the distribution of PRC units/pt in total

** denotes mean ± SD for the distribution of PRC units per transfused patient

◇ p < 0.001

◇◇ p < 0.0001

Transfusion data for the entire hospitalization are shown in Table 1. Overall, during hospital stay 137 patients were transfused, receiving 289 units of PRCs, and the total number of PRC units transfused was significantly lower in group A (113 units) compared to group B (176 units, p < 0.0001). The percentage of patients receiving PRC transfusions was significantly lower in group A (62 of 100 patients) compared to group B (75 of 92 patients, p < 0.001).

Intraoperatively, 81 patients were transfused, receiving 142 units of PRCs. The number of intraoperative PRC transfusions was significantly lower in group A (31 units) compared to group B (111 units, p < 0.0001), and the percentage of patients receiving intraoperative transfusions was significantly lower in group A (19 of 100 in group A, vs. 62 of 92 in group B, p < 0.0001).

In the ICU, 93 patients received a total of 147 PRC units, and the number of PRC transfusions was slightly, but not significantly higher in group A (82 units) compared to group B (65 units). Likewise, the percentage of patients receiving transfusions in the ICU was slightly higher in group A (51 of 100 in group A, vs. 42 of 92 in group B), but the difference was not significant.

Table 2 presents demographic and clinical OR and ICU data, after dividing study patients to those transfused and those not transfused. Transfused patients had significantly higher age, lower height, weight and BSA, and lower preoperative hematocrit compared to those not transfused, whereas BMI and discharge hematocrit did not differ significantly. Male gender and assignment to group A (restrictive protocol) were strongly (p < 0.003) associated with lower probability of transfusion (Table 3).

**Table 2:** Baseline demographic and clinical (OR and ICU) data on transfused (n = 137) and not transfused patients (n = 55).

	Group Statistics		Independent samples tests				
	
	Transfusion		Levene's test	t-test for equality of means (equal variances assumed)			
		
		mean ± SD	Sig.	Sig (2-tailed)	Mean differ.	Std error differ.	96% CI* lower/upper
Age	NO	63.6 ± 9.8	0.007	0.016	-3.09	1.27	-5.59/0.58
	YES	66.7 ± 7.1					
Weight	NO	79.3 ± 11.2	0.563	0.020	4.09	1.75	0.64/7.55
	YES	75.2 ± 10.9					
Height	NO	171.0 ± 6.2	0.12	0.000	4.94	1.19	2.60/7.29
	YES	166.1 ± 7.9					
BMI	NO	27.1 ± 3.2	0.442	0.745	-0.17	0.53	-1.22/0.88
	YES	27.2 ± 3.4					
BSA	NO	1.90 ± 0.15	0.949	0.001	0.90	0.02	0.04/0.14
	YES	1.81 ± 0.16					
pre-op. Hct	NO	42.1 ± 3.78	0.81	0.000	2.42	0.64	1.16/3.69
	YES	39.6 ± 4.10					
discharge Hct	NO	32.8 ± 2.21	0.829	0.586	-1.19	0.35	-0.87/0.49
	YES	33.0 ± 2.15					

* Confidence interval of the difference

**Table 3:** Results of Chi-square tests evaluating the association of Transfusion with Fluid administration protocol and Gender.

		Cross-tabs		Chi-square tests			
		**Transfusion**		**Asympt. Sig. (2-sided)**	**Exact. Sig. (2-sided)**	**Exact. Sig. (1-sided)**	
						
		**NO**	**YES**				

Fluid admin	Restricted	38	62	0.003			Pearson chi square
	Liberal	17	75		0.004	0.002	Fisher's exact test

Gender	Male	54	105	0.000			Pearson chi square
	Female	1	32		0.000	0.000	Fisher's exact test

Table 4 presents data after dividing patients within each group, in two subgroups, based on whether they received intraoperative PRC transfusions or not. Among patients transfused in the OR, significant difference existed between patients belonging in group A and B for gender, age and BSA (Table 4). Logistic regression modelling (Tables 5 &6) identified three variables as significant predictors of transfusion: fluid administration policy (group assignment), preoperative hematocrit and BSA (Table 5). The model explains nearly 21.5% (Nagelkerke R^2^, Table 6) of the observed variability regarding receiving a transfusion or not, and shows that the likelihood of PRC transfusion is 3.12 times greater in group B compared to group A. Furthermore, each 1% increase of preoperative hematocrit is associated with 15% (CI 5% - 26%) lower probability of transfusion.

**Table 4:** Patient data, with each patient group divided in two subgroups, based on whether patients were transfused in the operating room or not

Variable	Group A (fluid restriction)		Group B (liberal fluid administration)	
	
	Transfused (19 pts)	Not transfused (81 pts)	Transfused (62 pts)	Not transfused (30 pts)
Age (yr)	70.4 ± 4.76	65.0 ± 8.15	65.5 ± 7.42 ##	63.4 ± 9.59
Females, n (%)	8 (47.0%)	9 (52.9%)	14 (87.5%) ◇◇	2 (12.5%)
Weight (kg)	72.9 ± 12.12	78.2 ± 11.24	74.5 ± 9.30	77.4 ± 12.81
Height (cm)	160.3 ± 5.63	168.6 ± 7.38	166.5 ± 7.98 ◇	171.0 ± 6.40
BMI	28.4 ± 4.77	27.4 ± 3.17	26.8 ± 2.83	26.4 ± 3.59
BSA (m^2^)	1.73 ± 0.14	1.87 ± 0.17	1.81 ± 0.15 #	1.88 ± 0.17
Preop. Hct (%)	38.4 ± 2.86	40.6 ± 4.62	39.9 ± 3.77	41.9 ± 3.79

# p < 0.05, ## p < 0.01, ◇ p < 0.001, ◇◇ p < 0.0001, when comparing transfused Group A patients vs. Transfused Group B patients.

**Table 5:** Variables in the Logistic Regression Equation

								95% CI for EXP(B)	
								
		B	S.E.	Wald	df	Sig.	Exp(B)	Lower	Upper
Step	Group (A)	-1.137	0.364	9.767	1	0.002	0.321	0.157	0.654
1a	Pre-op Hct	-0.139	0.046	9.107	1	0.003	0.87	0.795	0.952
	BSA	-2.728	1.103	6.114	1	0.013	0.065	0.008	0.568
	Constant	12.320	2.571	22.957	1	0.000	224214.3		

a. Variable(s) entered on step 1: BSA

SE: Standard Error, df: degrees of freedom, CI: confidence interval

**Table 6:** Logistic Regression model summary

Step	-2 Log likelihood	Cox & Snell R Square	Nagelkerke R Square
1	198.839^a^	0.15	0.215

^a ^Estimation terminated at iteration number 5

**Figure 1:**
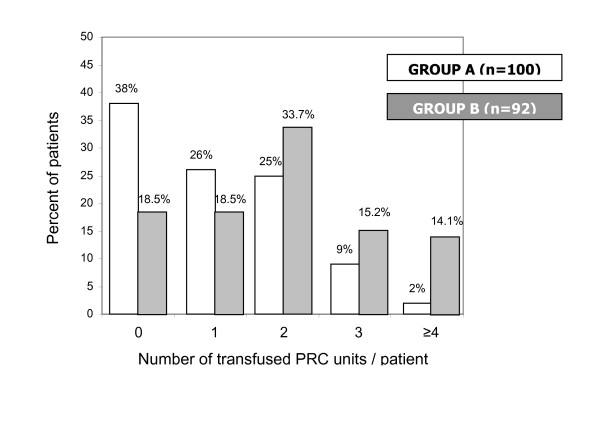
**Number of transfused PRC units/patient. Significantly more Group A patients received 0 or 1 PRC unit, whereas significantly more Group B patients received 3, 4, or more PRC units (p < 0.0007)**.

Results concerning the number of PRC units transfused per patient are displayed in Table 7 and graphically presented in Figure 1. Significantly more Group A patients received 0 or 1 PRC unit, whereas significantly more Group B patients received 3, 4 or more PRC units (p < 0.0007). Statistical analysis of the association between the two most significant parameters derived from logistic regression (group assignment and preoperative hematocrit) with the number of PRCu/pt could not reach any safe conclusions, but increased PRC u/pt negatively correlated to the number of patients receiving such transfusion in group A.

**Table 7:** Cross-tabulation of transfused PRC units per patient (combined OR and ICU data) by group.

PRC units per patient	GROUP A	GROUP B	TOTAL
0	38	17	55
1	26	17	43
2	25	31	56
3	9	14	23
≥4	2	13	15

TOTAL	100	92	192

Significantly more Group A patients received 0 or 1 units, whereas more Group B patients received 3, 4, or more units (p < 0.0007).

Table 8 shows hematocrit values for the entire observation period. Hematocrit decreased in both groups 10 minutes after CPB initiation and gradually increased towards discharge, presenting insignificant difference between groups at that point. Hematocrit values differed significantly between groups in sampling 3 (p < 0.05) and 4 (p < 0.005), but did not differ at any time during ICU stay. Data on fluid balance are also displayed in table 8. Only 9 of 100 group A patients received more than 500 ml of IV fluids before CPB. For this period, hydroxyethylstarch represented 95% of volume administered in group A but only 50% in group B, with the rest being crystalloid (not including saline for drug dialyses). Fluid administered in the period before CPB differed significantly between groups (p < 0.0001). Likewise, between CPB initiation and the first cardioplegia administration (sampling 4), fluid balance differed significantly between groups (p < 0.0001). Urine output and fluid balance while on CPB [ = (pump prime + total cardioplegia + any other "extra" volume in the CPB machine) - (urine + hemofiltration volume + residual CPB circuit volume)] are also displayed. Urine output did not differ between groups. Fluid balance for the entire procedure was significantly lower in group A (390 ± 432) compared to group B (667 ± 553, p < 0.001). Calculated net erythrocyte volume loss during the entire procedure was significantly lower in group A (758 ± 299 ml) compared to group B (903 ± 303 ml, p < 0.005).

**Table 8:** Hematocrit values and fluid balance by patient group.

HEMATOCRIT VALUES	Group A	Group B
1. Preoperative	40.21 ± 4.42	40.57 ± 3.87
2. After arterial line placement	39.59 ± 4.72	39.04 ± 4.41
3. After anesthesia induction	37.81 ± 4.69	36.44 ± 4.03#
4. After first cardioplegia	21.26 ± 3.49	19.96 ± 3.56#
5. End of CPB	24.53 ± 3.06	24.10 ± 2.30
6. End of operation	27.23 ± 3.20	26.46 ± 2.29
7. 6 hours in the ICU	28.98 ± 3.37	28.34 ± 2.49
8. 12 hours in the ICU	30.30 ± 2.79	30.67 ± 2.60
9. Day of discharge	32.74 ± 2.22	33.13 ± 2.09

**FLUID BALANCE**		
IV fluids (ml) to initiation of CPB	328 ± 157	642 ± 222◇◇
urine (ml) to initiation of CPB	141 ± 106	169 ± 111
fluid balance after 1^st ^cardioplegia	2058 ± 236	2323 ± 365◇◇
urine (ml) during CPB	822 ± 483	838 ± 378
total urine production (ml)	1455 ± 532	1538 ± 546
use of filter, n (%)	11 (11.0%)	20 (21.7%)##
Overall fluid balance	390 ± 432	667 ± 553 ◇

**Calculated erythrocyte volume loss**	758 ± 299	903 ± 303##

# p < 0.05

## p < 0.005

◇ p < 0.001

◇◇ p < 0.0001

There were no OR deaths in either group. Mechanical ventilation duration ranged from 5 to 52 hours (mean = 9.5, median = 9) in group A, and from 5 to 70 hours (mean = 13.2, median = 10) in group B. ICU LOS ranged from 1 to 10 days (mean = 2.6, median = 2) in group A, and from 1 to 8 days (mean = 3.2, median = 2) in group B. Mechanical ventilation duration and ICU LOS did not differ significantly between groups. Likewise, postoperative LOS in the ward did not differ between groups (8.4 ± 2.2 in group A vs. 8.1 ± 2.9 in group B). ICU complications included MI (5 pt), persistent significant arrhythmia (third-degree atrioventricular heart block, supraventricular tachyarrhythmias or symptomatic ventricular arrhythmias) (8 pts), low output syndrome delaying extubation (6 pts) and persistent neurological dysfunction (1 pt) in group A and MI (4 pt), arrhythmia (6 pts), low output syndrome (7 pt), and lower extremity ischemia (1 pt) in group B. Excluding patients with complications in the ICU, ventilation time >24 h occurred in 5 group A patients and 6 group B patients. Reoperation for bleeding occurred in one group A patient who had not been transfused during the initial operation, and one group B patient who had already been transfused during the initial operation. In total, re-explored patients received 4 and 6 PRC units respectively. One patient in each group developed renal failure and required dialysis. Finally, among patients with complications, two group A patients (one had CABG, one had AVR) and one group B patient (had CABG) died in the 30-day postoperative period.

## Discussion

Decisions regarding PRC transfusion are based on a multimodal approach in cardiac surgery, and the correct, if any, transfusion trigger remains contentious. We designed this study because we believe that fluid balance is a modifiable variable that can impact hematocrit and thereby influence the number of PRC units transfused. The study demonstrated reduced intraoperative PRC transfusion and less positive fluid balance in the "restricted fluid" group, while hematocrit values were not significantly different between groups at the end of the operation. Among patients who received intraoperative PRC transfusions, significantly fewer belonged to group A. Postoperatively, the number of transfused patients and the number of PRC units did not differ significantly between groups.

We propose that the lower transfusion rate in group A is attributable to our protocol, which was designed to avoid unnecessary fluid loading. Hematocrit and fluid balance differed significantly between groups after CPB and at the end of surgery, because group A patients received fluids only for hypovolemia, but not to compensate for vasodilatation or poor cardiac performance. Our study showed that relatively small differences in parenteral fluid administration can significantly influence intraoperative transfusion.

Strengths of this study include study design (prospective, randomized, adequate power). Use of a well-defined PRC transfusion protocol and having all operations performed by the same team under similar conditions makes the study stronger, and the low number of deaths resulted in data with few missing data points.

Study limitations include certain aspects of study design (no formal blinding, different anesthesiologists in different cases). Furthermore, our low mortality may reduce generalizability of the results, as our conclusions may not be applicable in cardiac surgery centers where more transfusions are needed because of higher surgical complication rates. In addition, lack of standardization with regards to intravenous fluid administration in group B (liberal fluids) is also a limitation. We believe that the observed difference between groups concerning replacement solutions probably resulted from use of a carrier fluid and from "liberal" fluid administration in group B. Unfortunately, this important difference between groups only became obvious during data analysis. However, we believe this important limitation is not necessarily a major drawback because, as group B patients received approximately 50% crystalloid and 50% colloid, both groups overall received similar amounts of colloid, and only differed in the amount of crystalloids given to group B.

Despite receiving more PRC units during CPB, group B patients had lower intraoperative Hct values (Table 8). In addition to hemodilution from liberal fluid administration, the observed differences between groups could also be attributed to variability in the transfusion trigger and variability in fluid administration during CPB between groups: The study protocol required that Clinicians in Group A consider more sophisticated data like INVOS values before initiating a blood transfusion, whereas group B patients were transfused at the discretion of the attending anaesthesiologist when Hct values were between 17-21%. Absence of a protocol for transfusion of other blood products (FFP, platelets, and cryoprecipitate) should also be pointed out as a weakness, because differences in treatment of coagulation abnormalities could result in greater variability of blood loss, and possibly of transfusions.

As advanced age, female gender, low BSA and preoperative anemia have been identified as independent predictors of PRC transfusion in cardiac surgery [5,7,8,11], blood loss and CPB initiation are expected to have a greater impact on hemoglobin concentration in these patient categories. Patients who received transfusions in our study differed significantly, compared to patients who were not transfused with regards to these variables. Logistic regression showed that fluid restriction is a significant factor, decreasing the probability of transfusion to 0.32. Likewise, low preoperative hematocrit was also identified as significant: the probability of transfusion in a patient with 36% preoperative hematocrit is almost twice the probability of a patient with preoperative hematocrit of 42%. Mean preoperative hematocrit was significantly lower in transfused patients compared to those not transfused (Table 2). In addition, among patients transfused in the OR, hematocrit in group A did not differ significantly compared to group B (Table 4). Consequently, preoperative anemia seems to predispose to transfusion even under a fluid restriction protocol. Subgroup analysis of our data could perhaps help us extract clear conclusions regarding specific population groups (e.g. low BMI patients). However, because our study did not have adequate power for subgroup analysis, appropriately designed rigorous clinical trials are needed to fully determine the effect of intra-operative fluid restriction in specific population groups.

Wide variations in reported transfusion practices [10,12] probably reflect variability between institutions, but also indicate that transfusion decisions have a degree of subjectivity [7,12]. It seems that we, as anesthesiologists, do not really know the degree of hemodilution that can be tolerated by each patient. A significant proportion of intraoperative transfusions occur during CPB, when SVO_2 _monitoring is impossible, and blood samples drawn from the venous cannula give an inconclusive picture about tissue oxygenation, because the heart is bypassed and hemoglobin saturation values are normalized by cold, less oxygen-consuming tissues. In our study, transfusion decisions during CPB were based on hematocrit value, clinical condition, INVOS data, time to release aortic clamp, temperature and urine production. We believe that two factors influenced transfusion decisions during this period: experience of the anesthesiologist (interpretation of the above parameters) and protocol. Less experienced anesthesiologists may have responded to excessive hemodilution (more likely in group B) with unnecessary transfusions. The strict INVOS-based protocol and the directions for using BIS data in group A may have also played a role, but the true value of INVOS with regards to transfusion decisions in cardiac surgery is unknown. For example, we do not know how to treat a patient with hematocrit less than 17% with normal INVOS values during CPB. Is transfusion justified at this point? Existing reports raise concerns regarding safety when proceeding with low hematocrit values [13,14]. In any case, low hematocrit values during CPB are associated with excessive hemodilution. Finally, BIS data may have prompted the anesthesiologist to intervene directly or indirectly to aspects of patient care other than hypnotic state depth [15].

The observed difference of calculated erythrocyte volume loss between the two groups deserves comment, because blood loss affects transfusion decisions. First, this difference is difficult to explain, because the two groups originated from randomization, had similar baseline data, were operated under exactly the same conditions, and surgery duration did not differ significantly between groups. Second, erythrocyte volume loss calculations are based on formulas taking into account preoperative patient data. Consequently, because allogeneic red cells can be displaced from the circulation earlier than native erythrocytes, erythrocyte volume loss can be overestimated as the number of transfused units increases. In any case, we certainly have some reservation regarding the validity of these methods.

Outcome data, other than PRC transfusions, did not differ significantly between groups in our study. However, this study was designed to compare PRC transfusions between groups, and did not have the power to show differences with regards to other important outcomes, such as renal failure, length of stay, morbidity or mortality. Because such comparisons are beyond the size and scope of our study, we believe that convincing answers to these important questions can only come from well designed future studies with much larger patient populations.

## Conclusions

The results of this study show that intraoperative IV fluid restriction combined with red cell salvage and a well-defined PRC transfusion protocol reduces intraoperative PRC transfusion in cardiac surgery without significantly increasing postoperative PRC transfusion. The benefits of fluid restriction are more pronounced in patients prone to transfusion (such as aged females, patients with low BSA or low preoperative hematocrit). Current evidence suggests that physician transfusion practices can be improved. Consequently, appropriately designed rigorous clinical trials are needed to confirm the validity of our findings and determine the combined effectiveness of new monitoring modalities and intraoperative fluid restriction on blood conservation, and their role on rational decision-making regarding PRC transfusion in cardiac surgery.

## List of Abbreviations

ACT: activated clotting time; AOX: aortic cross-clamping; ASD: atrial septal defect; AVR: aortic valve replacement; BIS: bispectral index; BMI: body mass index; BSA: body surface area; CABG: coronary artery bypass grafting; CI: confidence interval; COPD; chronic obstructive pulmonary disease; CPB: cardio-pulmonary by pass; ECG: electrocardiogram; Hct: hematocrit; ICU: Intensive Care Unit; INVOS: near infrared spectroscopy; IV: intravenous; LOS: length of stay; LVEF: left ventricular ejection fraction; MI: myocardial infarction; MAP: mean arterial pressure; MVR: mitral valve replacement; NYHA: New York Heart Association; OR: Operating Room; PCWP: pulmonary capillary wedge pressure; PRC: packed red cells; RCT: randomized control trial; SD: standard deviation; SvO_2_: mixed venous oxygen saturation.

## Competing interests

This research project was supported solely by department funds. All authors declare they have no conflict of interest to report

## Authors' contributions

All authors: 1) have made substantial contributions to conception and design of the study or acquisition of data, or analysis and interpretation of data; 2) have been involved in drafting the manuscript or revising it critically for intellectual content; and 3) have approved the final version to be published.
